# Perimenopausal syndrome and hypertension during perimenopause in South China: prevalence, relationships and risk factors

**DOI:** 10.1186/s12905-024-03056-5

**Published:** 2024-04-03

**Authors:** Zheng Li, Jun-Ping Guo, Liu Huang

**Affiliations:** 1 Obstetrics and Gynecology, Wuyunshan Hospital of Hangzhou, Hangzhou, 310000 China; 2 Medical School, Hangzhou Normal University, Hangzhou, 310000 China

**Keywords:** Hypertension, Perimenopause syndrome, Prevalence, Relationship, Factors

## Abstract

**Background:**

More than 2 billion women are experiencing the menopausal transition in China, and some of these women have hypertension. Limited studies has focused on perimenopausal syndrome and hypertension in a specific population, so we aimed to investigate the prevalence of perimenopausal syndrome and hypertension and to analyse their relationships and risk factors in perimenopausal women in South China.

**Methods:**

This cross-sectional study included 3553 women aged 40 to 60 years from South China. We collected medical report, lifestyle, blood sample, general condition questionnaire, and modified Kupperman index (mKMI) data. Multivariate logistic regression analysis was performed to identify risk factors for perimenopausal syndrome and hypertension during perimenopause.

**Results:**

The prevalence of hypertension in perimenopause patients was 16.58%, and the prevalence of perimenopausal syndrome was 9.9%. Compared with women without hypertension during perimenopause, women with HTN during perimenopause had an increased risk of perimenopausal syndrome (26.4% vs. 8.7%, *P* < 0.001). Lipid levels and urinary tract infections were risk factors for hypertension and perimenopausal syndrome, in addition to the presence of breast nodules, the intake of snacks at night, high-salt diets, red meat and sugar-sweetened beverages, and a history of smoking and drinking for perimenopausal syndrome and the presence of gestational hypertension and diabetes for hypertension.

**Conclusion:**

We concluded that perimenopausal syndrome and HTN are common in perimenopausal women in South China, and the associations between them are strong and positive. Perimenopausal syndrome shares some common risk factors with HTN during perimenopause, such as BMI and dyslipidaemia. Therefore, gynaecological endocrinologists in China should consider screening for perimenopausal syndrome in hypertensive perimenopausal women, and appropriate management of perimenopause is needed to alleviate these conditions.

## Background

Perimenopause refers to the period when a woman develops endocrinological, biological and clinical features associated with menopause up to 1 year after her last menstrual cycle, which occurs between the ages of 40 and 60 years [1]. According to a survey conducted by the World Health Organization (WHO) in 2012, the number of perimenopausal women is expected to reach 1.2 billion in 2030, and nearly 76% of these women will live in developing countries [2]. Recent studies in China revealed that the number of perimenopausal women has reached 120 million, the largest among all developing countries, accounting for 10% of the Chinese population and 23% of the total number of perimenopausal women worldwide [3]. Perimenopausal syndrome refers to a series of autonomic nervous system dysfunctions and neuropsychological symptoms, such as hot flashes, menstrual changes, emotional fluctuations, fatigue, insomnia, and bone and joint pain, which are caused by fluctuations or decreases in sex hormone levels during the perimenopausal period. These changes can cause a series of physiological and psychological problems, seriously affect normal life and work, and even cause family discord and other social problems. The incidence of perimenopausal syndrome in China is 68.1% [4].

Hypertension (HTN) is the main cardiovascular risk factor, affecting 25% of women, and this issue is especially important when women with high blood pressure reach menopause. Cross-sectional data have shown a fourfold greater incidence of hypertension in postmenopausal women than in premenopausal women [5]. Hypertension after menopause may lead to increased risk of target organ damage and cardiovascular disease, such as increased arterial stiffness, coronary disease, chronic heart failure and stroke [6]. Therefore, hypertension in postmenopausal women is often associated with cardiovascular risk factors such as a high BMI, dyslipidaemia, chronic low-grade inflammation, oxidative stress, endothelial dysfunction, and cardiac remodelling [7–9].

Studies have shown that women have lower blood pressure than men of the same age until middle age, whereas the reverse seems to occur thereafter; in other words, fertile women are protected from cardiovascular risk by circulating oestrogens, a protective factor that is lost during menopause. After menopause, women are not different from men from the point of view of risk factors and cardiovascular events [10]. Menopause is commonly associated with an increase in blood pressure (BP); however, cross-sectional or longitudinal studies conducted thus far have often been too limited and have been unable to indicate whether this increase in BP is truly dependent on menopause or caused by age or changes in body mass index (BMI). If this hypothesis is well founded, menopause can be regarded as one of the major, as well as the most ineluctable, risk factors for cardiovascular disease, affecting more than half of the population,. Therefore, we conducted a study that focused on the relationship between HTN and perimenopausal syndrome to provide a theoretical reference for the epidemiology, prevention and control of perimenopausal syndrome in women.

## Methods

### Subjects

For this cross-sectional study, the data of 3553 women (40–60 years old) who underwent routine physical examinations in Zhejiang Province from October 2018 to October 2021 were reviewed. The inclusion criteria were as follows: (1) no previous history of thyroid, breast, or uterine cancer surgery; (2) no missing physical examination data; and (3) agreed to participate in the study. The exclusion criteria were as follows: (1) the onset of menopause due to surgical removal of the ovaries and uterus for various reasons; (2) mental illness, hearing impairment or cognitive impairment; and (3) secondary hypertension. The study was approved by the Medical Ethics Committee of Hangzhou WuYunShan Hospital (No. 2,020,001) and was carried out in accordance with relevant guidelines and regulations. Informed consent was obtained from all participants.

### Demographic information and epidemiological investigation

Demographic characteristics, lifestyle information, physiological-biochemical indices and other anthropometric data were collected in this study. Demographic characteristics and lifestyle information were collected through a semiquantitative questionnaire. The main lifestyle information was as follows: (1) the intake of a high-salt diet were defined as a diet with high salt intake; (2) the intake of milk/fruit/fish/coffee/eggs was defined as “<3 times/week” or “≥3 times/week”. To evaluate perimenopausal syndrome, we used the modified Kupperman index (mKMI); this index is widely used internationally, including in the People’s Republic of China, and its role in clinical practice is well established [11, 12]. The scale comprises 13 items: the weighted score for hot flashes or sweating is 4 points; paraesthesia, insomnia, mood swings, sexual problems, and urinary infection receive 2 points each; and other symptoms receive 1 point each [13]. mKMI scores were divided into 4 grades: 0 (none), 1 (mild), 2 (moderate), and 3 (severe). The sum of the scores of all the items is the total Kupperman score. Perimenopausal syndrome is considered mild if the total Kupperman score is 15 to 24 points, moderate if the score is 25 to 34 points, and severe if the score is greater than 34 points.

### Definition

Hypertension was defined as a systolic blood pressure (SBP) ≥ 140 mmHg and/or a diastolic blood pressure (DBP) ≥ 90 mmHg or self-reported current antihypertensive medication use; general overweight was defined as a BMI ≥ 24 kg/m2 according to Chinese standards; abdominal overweight or obesity was defined as a WC ≥ 80 cm; and the cut-off points for dyslipidaemia were a TG level ≥ 1.7 mmol/L, a TC level ≥ 5.2 mmol/L, an LDL-C level ≥ 3.4 mmol/L, an HDL-C level < 1.0 mmol/L, a non-HDL-C level ≥ 4.1 mmol/L, and a TC/HDL-C ratio ≥ 5.0 mmol/L. BMI was classified as follows: 18.5 ≥ body mass index (BMI) < 24 kg/m2 was defined as normal; 24 ≥ BMI < 28 kg/m2 was defined as overweight; and a BMI ≥ 28 kg/m2 was defined as obese. Ultrasonography was performed by the same experienced sonographer, and the diagnosis of thyroid nodules, uterine fibroids (UFs) and urinary tract infections was determined by international standards [14–16].

### Statistical analysis

Statistical analysis was conducted with SPSS 24.0 software and the glmnet package of RStudio (version 1.1.456). The chi-squared test, Fisher’s exact test (for categorical variables), t tests, and the Wilcoxon rank sum test (for continuous variables) were used to evaluate demographic characteristics. The odds ratios (ORs) and 95% confidence intervals (CIs) determined by logistic regression analysis were used to analyse the associations between potential risk factors and HTN or perimenopausal syndrome. In all analyses, p values < 0.05 were considered to indicate a statistically significant difference.

## Results

### General characteristics

Table 1 shows the basic characteristics of the 3553 individuals in the case/control groups. The average age of the patients in the case/control group was 50.96 ± 4.62/51.01 ± 5.20 years, and there was no significant difference in the age distribution between the two groups. There were statistically significant differences between the two groups with regard to weight, BMI, WC, TG level, HDL-C level, LDL-C level and some lifestyle factors (the intake of milk, eggs, fruit, fish and coffee) (all *p* > 0.05). The prevalence of HTN in perimenopause patients was 16.58%.

**Table 1:** Basic characteristics of perimenopausal women

Characteristics	Participants with hypertension	Participants without hypertension	t/z/χ2	P value
Total	589	2964		
Age, Median (IQR), years	50.96(7)	51.01(8)	0.13^t^	0.7167
Weight, Median (IQR), kg	61.43(11.55)	57.34(9.5)	-12.41^t^	< 0.001
BMI, mean ± SD, kg/m2	22.65 ± 2.48	24.32 ± 3.04	-14.26^t^	< 0.001
WC, Median (IQR), cm	79.86(11)	74.93(9)	-15.63^t^	< 0.001
TC, Median (IQR), mmol/L	5.091.21)	4.90(4.52)	-5.09^t^	< 0.001
TG, Median (IQR), mmol/L	1.75(0.98)	1.30(0.73)	-11.18^t^	< 0.001
HDL-C, Median (IQR), mmol/L	1.45(0.44)	1.52(0.45)	4.45^t^	< 0.001
LDL-C, Median (IQR), mmol/L	3.02(0.93)	2.83(0.92)	-6.18^t^	< 0.001
Hypertension of pregnancy, n (%)				
+	39(6.7)	64(2.2)	34.30^χ2^	< 0.001
	545(93.3)	2885(97.8)		
Dyslipidaemia, n (%)				
+	382(64.9)	1355(45.7)	72.04^χ2^	< 0.001
	207(35.1)	1609(54.3)		
History of diabetes, n (%)				
+	42(7.10)	48(1.60)	60.45^χ2^	< 0.001
	547(92.9)	2916(98.4)		
Gestational diabetes, n (%)				
+	119(1.9)	76(2.6)	1.042^χ2^	0.307
	573(98.1)	2843(94.4)		
Uterine fibroids, n (%)				
+	304(51.6)	1359(45.9)	6.55^χ2^	0.010
	285(48.3)	1605(54.1)		
Thyroid nodules, n (%)				
+	426(72.3)	1913(64.5)	13.24^χ2^	< 0.001
	163(27.7)	1051(35.5)		
Urinary tract infection, n (%)			7.49^χ2^	0.006
+	204 (34.6)	859 (29.0)		
	385 (65.4)	2105 (71.0)		
High-salt diets, n (%)			6.33^χ2^	0.012
+	107 (18.2)	419 (14.1)		
	482 (81.8)	2545 (85.9)		
Intake of milk, n (%)			4.45^χ2^	0.035
< 3 times/week	112 (19.0)	681 (23.0)		
≥ 3 times/week	477 (81.0)	2283 (77.0)		
Intake of eggs, n (%)				
Hardly ever	45(7.6)	133(4.5)	10.26^χ2^	0.001
Regularly (≥ 2 times/week)	544(92.4)	2831(95.5)		
Intake of fruit, n (%)				
< 3 times/week	182(30.9)	793(26.8)	4.24^χ2^	0.039
≥ 3 times/week	407(69.1)	2171(73.2)		
Intake of the flesh of fish, n (%)				
< 3 times/week	352(59.8)	1627(54.9)	4.72^χ2^	0.030
≥ 3 times/week	237(40.2)	1337(45.1)		
Intake of coffee, n (%)				
Never	419(71.1)	1981(66.8)	-1.95^z^	0.051
< 3 times/week	154(26.1)	906(30.6)		
≥ 3 times/week	16(2.7)	77(2.6%)		
Feels down in the dumps, n (%)				
Never	464(73.7)	2153(72.6)	-3.19^z^	0.001
Sometimes	120(20.4)	749(25.3)		
Frequently	5(0.8)	62(2.1)		
Feels heated with passion, n (%)			-2.82^z^	0.005
Never	401(68.1)	1840(62.1)		
Sometimes	167(28.4)	979(33.0)		
Frequently	21(3.6)	145(4.9)		
Feels as if on thorns, n (%)			-2.44^z^	0.015
Never	460(78.1)	2180(73.5)		
Sometimes	120(20.4)	690(23.3)		
Frequently	9(1.5)	94(3.2)		

WC, waist circumference; BMI, body mass index; TC, total cholesterol; TG, triglyceride; IQR, interquartile range; SD, standard deviation; HDL-C, high-density lipoprotein cholesterol; LDL-C, low-density lipoprotein cholesterol; t, t test; z, Wilcoxon rank sum test; χ2, chi-squared test. A p value < 0.05 was considered to indicate statistical significance

### Prevalence of perimenopausal syndrome and perimenopausal symptoms

Table 2 shows that the prevalence of perimenopausal syndrome varied by age, and these differences were statistically significant (χ2 = 8.57, *P* = 0.036). Table 3 shows the proportions of patients with each perimenopausal symptom. The study revealed that the prevalence of perimenopausal syndrome was 9.9% (Table 2), but more than 95% of the participants had at least one symptom (Table 3). The top five typical symptoms of perimenopausal syndrome were insomnia (56.9%), fatigue (45.6%), sexual problems (37.5%), mood swings (37.3%), and vertigo (34.2%). Table 3 also shows the relationships between perimenopausal syndrome and hypertension. The prevalence of perimenopausal syndrome and many perimenopausal symptoms in the hypertension group was significantly greater than that in the nonhypertensive group (*P* < 0.001).

**Table 2:** Prevalence of perimenopausal syndrome

Age (years)	Nonperimenopausal syndrome (n,%)	Perimenopausal syndrome (n,%)	Total	
40–45	518 (89.01))	64(19.9)	582	
46–50	697(89.82)	79(10.18)	776	
51–55	1120(92.03)	97(7.97)	1217	
56–60	866(88.54)	112(11.45)	978	
Total	3201	352	3553	
χ2	8.57			
P	0.036			

^a^ Perimenopausal syndrome is defined as an mKMI score ≥ 15; mKMI, modified Kupperman index

**Table 3:** Frequency of perimenopausal symptoms in perimenopause patients

Symptoms	HTN (N,%)	Non-HTN (N,%)	Total (N,%)	χ2	P
Hot flashes/sweating	132(22.4)	496(16.7)	628(17.7)	26.04	<0.001
Urinary tract infection	206(35.0)	859(29.0)	1065(30.0)	13.23	0.001
Insomnia	521(89.5)	1503(51.7)	2024(56.9)	309.38	<0.001
Headache	149(25.3)	730(24.62)	879 (24.7)	0.18	0.73
Sexual problems	343(58.2)	990(33.4)	1333(37.5)	146.52	<0.001
Paraesthesia	83(14.1)	507(17.1)	590(16.6)	3.22	0.073
Fatigue	263(44.7)	1357(45.8)	1620(45.6)	2.56	0.47
Palpitations	148(25.1)	740(25.1)	888(25.0)	0.038	0.845
Muscle/joint pain	307(52.1)	777(26.2)	1084(30.5)	185.97	<0.001
Vertigo	588(99.8)	627(21.1)	1215(34.2)	1354.0	<0.001
Melancholia	78(13.2)	614(20.7)	692(19.50)	21.88	<0.001
Mood swings	196(33.3)	1128(38.1)	1324(37.3)	8.70	0.034
Formication	35(5.9)	45(1.5)	80(2.3)	44.91	<0.001
Perimenopausal syndrome (mKMI score ≥ 15)	93(26.4)	259(8.7)	352(9.9)	27.37	<0.001
Total	589	2964			

^a^ mKMI, modified Kupperman index

^b^p value < 0.05 was considered to indicate statistical significance

### Risk factors for perimenopausal syndrome and hypertension in perimenopause patients

Univariate regression analysis revealed that, in addition to a history of gestational diabetes, all environmental factors were significantly associated with HTN during perimenopause. Among these factors, a high waist circumference, a high BMI, dyslipidaemia status, a history of hypertension during pregnancy, diabetes status, uterine fibroid status and the intake of a high-salt diet were risk factors, while the regular intake of fruit, vegetables, milk and fish was a protective factor (*P* < 0.01). After adjusting for other variables in the multivariate regression analysis, the main outcomes showed partial changes: the ORs changed; a history of gestational diabetes mellitus was a risk factor for hypertension; and the associations among the intake of a high-salt diet, the regular intake of fish, milk, and fruit and hypertension were no longer statistically significant (*P* > 0.05) (Table 4).

In regard to perimenopausal syndrome, Table 5 shows that in the univariate logistic regression, a high BMI, dyslipidaemia status, urinary tract infection status, the intake of late night snacks, the intake of a high-salt diet, the intake of red meat, long-term smoking status, passive smoking status and long-term alcohol consumption were risk factors (OR > 1, *P* < 0.05), while the consumption of three meals on time, regular exercise status, and the regular intake of fruit, vegetables and milk were protective factors (OR < 1, *P* < 0.05). After adjusting for other variables, most of the results remained the same; only the ORs changed, but the associations among late night snack consumption, fruit consumption and perimenopausal syndrome were no longer statistically significant (*P* > 0.05).

**Table 4:** Associations of environmental factors with the risk of HTN in perimenopause patients

Variables	Simple Logistic Regression Analysis			Multiple Logistic Regression Analysis		
	β	Unadjusted OR (95% CI)	P Values	β	Adjusted OR (95% CI)	P Values
WC	1.21	3.36 (2.80–4.03)	< 0.001	0.63	1.88 (1.49–2.37)	< 0.001
BMI						
overweight (24 - 27.9 kg/m^2^)	0.99	2.70 (2.23–3.27)	< 0.001	0.56	1.76 (1.40–2.21)	< 0.001
obese (≥ 28 kg/m^2^)	1.78	5.91 (4.20–8.31)	< 0.001	0.97	2.59 (1.73–3.87)	< 0.001
Dyslipidaemia	0.79	2.19 (1.82–2.63)	< 0.001	0.56	1.77 (1.45–2.15)	< 0.001
Hypertension during pregnancy	1.17	3.21 (2.14–4.83)	< 0.001	1.06	2.92 (1.86–4.61)	< 0.001
History of diabetes	1.54	4.67 (3.05–7.12)	< 0.001	1.13	3.17 (1.99–5.05)	< 0.001
Gestational diabetes	0.33	1.39 (0.73–2.64)	0.310	0.84	2.32 (1.15–4.71)	0.019
Uterine fibroids	0.23	1.26 (1.05–1.50)	0.011	0.25	1.28 (1.06–1.55)	0.010
Thyroid nodules	0.36	1.43 (1.18–1.75)	< 0.001	0.29	1.34 (1.09–1.65)	0.006
Urinary tract infection	0.26	1.30 (1.08–1.57)	0.006	0.22	1.24 (1.02–1.52)	0.034
Intake of a high-salt diet	0.30	1.35 (1.07–1.70)	0.012	0.11	1.12 (0.87–1.44)	0.383
Intake of fruit	-0.20	0.82 (0.67–0.99)	0.040	-0.06	0.95 (0.76–1.17)	0.608
Intake of milk	-0.24	0.79 (0.63–0.98)	0.035	-0.12	0.89 (0.70–1.13)	0.348
Intake of eggs	-0.56	0.57 (0.40–0.81)	0.002	-0.57	0.56 (0.39–0.83)	0.003
Intake of fish	-0.20	0.82 (0.68–0.98)	0.030	-0.13	0.89 (0.72–1.07)	0.194

WC (waist circumference) was classified as normal (< 80 cm) or out of limits (≥ 80 cm); BMI (body mass index) was classified as normal (18.5  -  23.9 kg/m^2^), overweight (24  -  27.9 kg/m^2^) or obese (≥ 28 kg/m^2^); the intake of eggs was classified as < 3 eggs per week or ≥ 3 eggs per week; and the intake of milk/fruit/fish was classified as < 3 times per week or ≥ 3 times per week

A p value < 0.05 was considered to indicate statistical significance

OR, odds ratio; CI, confidence interval

**Table 5:** Associations of environmental factors with the risk of perimenopausal syndrome

Variables	Simple Logistic Regression Analysis			Multiple Logistic Regression Analysis		
	β	Unadjusted OR (95% CI)	P Values	β	Adjusted OR (95% CI)	P Values
BMI						
overweight (24 - 27.99 kg/m^2^)	1.56	1.56(1.23–1.98)	< 0.001	0.26	1.30 (0.99–1.69)-	0.052
obese (≥ 289 kg/m^2^)	1.87	1.87(1.17–2.97)	< 0.001	0.34	1.40 (0.84–2.35)	0.199
Dyslipidaemia	0.50	1.65 (1.26–2.17)	< 0.001	0.56	1.75 (1.30–2.35)	< 0.001
History of diabetes	-0.44	0.64 (0.28–1.48)	0.30	-0.82	0.43 (0.18–1.07)	0.070
Uterine fibroids	0.03	1.14(0.91–1.41)	0.249	0.25	1.03 (0.81–1.31)	0.789
Thyroid nodules	0.36	0.95 (0.75–1.20)	0.659	-0.19	0.83 (0.64–1.06)	0.142
Breast nodules	0.22	1.25(0.99–1.56)	0.051	0.26	1.29(1.01–1.67)	< 0.001
Urinary tract infection	1.18	3.25 (2.60–4.07)	< 0.001	1.28	3.59 (2.84–4.55)	0.034
Intake of snacks at night						
Hardly (≤ 2 days/week)						
Frequently(3–5 days/week)	0.64	1.90(1.60–2.24)	< 0.001	0.31	1.36(0.99–1.86)	0.051
almost every day(≥ 6 days/week)	0.98	2.66(1.15–6.18)	0.022	0.50	1.65(0.65–4.17)	0.288
Three meals on time						
Hardly (≤ 2 days/week)						
Frequently(3–5 days/week)	-0.13	0.26(0.17–0.38)	< 0.001	-1.11	0.33(0.21–0.51)	< 0.001
almost every day(≥ 6 days/week)	-0.81	0.45(0.29–0.68)	< 0.001	-0.63	0.53(0.34–0.84)	0.006
Intake of a high-salt diet	0.65	1.92 (1.47–2.51)	< 0.001	0.33	1.39(1.03–1.87)	0.03
Intake of fruit	-0.40	0.66 (0.53–0.84)	0.001	-0.22	0.81(0.62–1.05)	0.106
Intake of milk	-0.57	0.56 (0.41–0.76)	< 0.001	-0.45	0.64(0.45–0.88)	0.006
Intake of eggs	-0.20	0.81 (0.50–1.30)	0.387	-0.05	0.95(0.57–1.58)	0.844
Intake of vegetables	-0.61	0.54(0.39–0.75)	< 0.001	-0.38	0.69(0.48–0.99)	0.042
Intake of red meat	0.52	1.68(1.22–2.31)	0.001	0.52	1.68(1.19–2.38)	0.004
Intake of sugar-sweetened beverages	0.52	1.68(1.33–2.10)	< 0.001	0.28	1.33(1.03–1.71)	0.027
Intake of coffee	-0.19	0.82(0.51–1.32)	0.422	-0.38	0.69(0.42–1.13)	0.140
Smoking status	0.19	1.20(1.07–1.36)	0.003	0.18	1.20(1.04–1.37)	0.011
Drinking status	0.35	1.42(1.07–1.87)	0.014	0.34	1.40(1.03–1.90)	0.030
Exercise status	-0.57	0.57(0.46–0.71)	< 0.001	-0.39	0.68(0.53–0.87)	0.002

BMI (body mass index) was classified as normal (18.5  -  23.9 kg/m^2^), overweight (24  -  27.9 kg/m^2^) or obese (≥ 28 kg/m^2^); the intake of eggs was classified as < 3 eggs per week or ≥ 3 eggs per week; the intake of milk/fruit/fish was classified as < 3 times per week or ≥ 3 times per week; exercise status was classified as almost never (< 3 times per week) or regularly (≥ 3 times per week); smoking status was classified as never, smoking or passive smoking every day; and drinking status was classified as almost never or frequently (≥ 2 times per week)

A p value < 0.05 was considered to indicate statistical significance

OR, odds ratio; CI, confidence interval

## Discussion

In the present study, we explored the prevalence of perimenopausal syndrome and hypertension in South China to elucidate the relationships between these conditions and to determine the risk factors for both conditions. We found that the prevalence of perimenopausal syndrome and HTN was 9.9% and 16.57%, respectively. The differences in the incidence of perimenopausal syndrome among the different age groups were statistically significant. The relationships between perimenopausal syndrome and HTN were strong and positive. Many common risk factors are associated with and shared between perimenopausal syndrome and HTN.

We found that many studies focused on perimenopausal symptoms, but few studies focused on perimenopausal syndrome. Nonetheless, we found some data from limited studies. A survey including 2100 registered nurses aged 40 to 55 years from 20 hospitals in Beijing showed that 37.83% of the participants who experienced perimenopause had perimenopausal syndrome, and a survey including 1062 perimenopausal women aged 40 to 60 years showed that 10.92% of the participants had perimenopausal syndrome [17, 18]. This value was higher than that in our study, possibly because the population included in our study was mostly high-income (our hospital is a characteristic hospital focusing on physical examinations and cadre treatment and recuperation, and the service subjects are mainly in-service and retired cadres and senior customers of insurance companies whose incomes are generally higher than that of the general population; low income has long been considered a risk factor for menopausal syndrome [4]) and the work environment (the work environment in hospitals may be more stressful [18]). However, further studies are needed to determine the relationship between these conditions. Additionally, we also revealed that the prevalence of perimenopausal syndrome was higher in patients with hypertension than in patients without hypertension.

In our study, we found strong positive correlations between menopausal symptoms and hypertension during perimenopause. Clinical evidence has shown that the perimenopausal period is a risk factor for cardiovascular disease, including hypertension, in women [19]. An example of this phenomenon is the greater prevalence of hypertension in men between 30 and 45 years of age than in women of similar age, and the prevalence of hypertension in women after this age increases to a level similar to or exceeding that of men [20, 21]. Although it is widely accepted that the prevalence of hypertension during perimenopause is a natural part of ageing, the mechanism of this association has not been established. Many studies believe that the increase in blood pressure during perimenopause is mainly due to the loss of sex steroids, indicating that oestrogen has a protective effect against increased blood pressure [19, 22]. However, sex steroids play an important role in the development of perimenopausal syndrome [23], which may be one of the explanations for the link between hypertension during perimenopause and perimenopausal syndrome in our study. This might also be explained by the fact that many of the 13 symptoms of perimenopausal syndrome are also possible complications of hypertension or risk factors for hypertension, such as obvious mood swings and urinary system infections.

Many factors were found to be associated with perimenopausal syndrome and hypertension, although they were different. Excluding the items contained in the perimenopausal syndrome calculation entry, the present survey suggested that dyslipidaemia status, breast nodule status, the intake of snacks at night, high-salt diets, red meat and sugar-sweetened beverages, and a history of smoking and drinking are risk factors for perimenopausal syndrome, but the consumption of three meals on time, the regular intake of milk and vegetables, and regular exercise are protective factors, which means that it is important for women in perimenopause to maintain a healthy lifestyle. Advocating for and maintaining a healthy lifestyle is the core of health management and health education, which is also one of the important purposes of this study. The few previous studies on the risk factors for perimenopausal syndrome have generally attempted to determine whether perimenopausal syndrome is related to the workplace environment, environmental stress, mental state or income level, while few studies have focused on the correlation between perimenopausal syndrome and physiological or biochemical data or even diseases. Our study focused on the main influencing factors, including personal characteristics (such as height, weight, etc.), physiological and biochemical data (such as blood pressure, blood lipid levels, blood glucose levels, blood urine creatinine levels, and routine urine data), image data (such as ultrasonography of the thyroid/breast/uterus), and lifestyle information (such as diet and exercise preferences), which have rarely been investigated in previous studies of perimenopause.

We found that hypertension during perimenopause is associated with waist circumference, BMI, dyslipidaemia, gestational hypertension, diabetes, uterine fibroids, thyroid nodules, and urinary tract infections, which is consistent with previous research [24–30]. The results of our study revealed that the intake of a high-salt diet, fruit, milk and fish were related to HTN during perimenopause without adjusting for other variables. The intake of a high-salt diet was a risk factor, and an appropriate intake of vegetables, fruits, milk and fish was a protective factor, which was similar to the results of previous studies. However, after adjusting for other variables, the difference was not statistically significant for two reasons. On the one hand, this may be related to the sample size of the study, and on the other hand, it may be because most of the lifestyle-related answers were obtained by questioning the patients, and there may be recall bias. The association between hypertension and the intake of eggs has been studied extensively, with inconsistent results [31]. In the present study, moderate egg intake was found to be a protective factor against hypertension in women during perimenopause.

Interestingly, our study suggested that HTN during perimenopause is associated with urinary tract infections, which may be explained by the fact that the participants in our study were women in perimenopause who are particularly vulnerable to infections caused by urogenital tract atrophy. This may be explained by the fact that urinary tract infection is an item of the mKMI, which is used to determine the presence of menopausal syndrome. This result explains some of the results of this study from another perspective, namely, the strong correlation between menopausal syndrome and hypertension during menopause. Among these risk factors, dyslipidaemia and obesity were shared risk factors for perimenopausal syndrome and HTN in perimenopause patients.

Therefore, it is necessary to take appropriate measures to reduce the risk of perimenopausal syndrome and cardiovascular and cerebrovascular diseases, including hypertension, in women. Our study has several strengths. First, our study is one of the few studies to comprehensively measure the prevalence, relationships, and risk factors for perimenopausal syndrome and hypertension during perimenopause. Second, the prevalence, relationships, and risk factors for perimenopausal syndrome and hypertension were described for the first time in the same population. Third, some important new findings were revealed, for example, the associations between the mKMI score and hypertension and between some risk factors and perimenopausal syndrome and hypertension. Therefore, it is necessary to take appropriate measures to reduce the risk of perimenopausal syndrome and cardiovascular and cerebrovascular diseases, including hypertension, in women. As the first health care providers for perimenopausal women, gynaecologists should not only focus on the treatment of menopausal symptoms but also be concerned about other cardiovascular and cerebrovascular risks, including HTN. These conclusions provide a reference for the epidemiology and prevention of these conditions in women during perimenopause.

The present study also has several limitations. First, this was a cross-sectional survey, so we were unable to determine a cause and effect relationship of this association. In addition, data derived from the semiquantitative questionnaire were limited by self-reported data; therefore, recall bias should be considered. Moreover, the sample size was small, so a larger, multipopulation randomized study to verify this conclusion is needed.

## Conclusions

In conclusion, perimenopausal syndrome and HTN are common in perimenopausal women in South China, and the associations between them are strong and positive. Many risk factors are associated with and shared between perimenopausal syndrome and HTN in perimenopause, especially some lifestyle factors. Therefore, gynaecological endocrinologists in China should consider screening for perimenopausal syndrome in hypertensive perimenopausal women, and appropriate management of perimenopause is needed to alleviate these conditions. These results provide a theoretical basis for the health management of women and risk factor screening to prevent HTN and perimenopausal syndrome during perimenopause.

## Data Availability

The datasets analysed during the present study are not publicly available [the data are being further analysed] but are available from the corresponding author, J.P.G., upon reasonable request.

## Abbreviations

HTN: hypertension
BMI: body mass index
SBP: systolic blood pressure
DBP: diastolic blood pressure
TG: triglyceride
HDL-C: high-density lipoprotein cholesterol
LDL-C: low-density lipoprotein cholesterol
OR: odd ratio
CIs: confidence intervals
mKMI: modified Kupperman index
